# HIV knowledge, stigma, and illness beliefs among pediatric caregivers in Ghana who have not disclosed their child's HIV status

**DOI:** 10.1080/09540121.2015.1007116

**Published:** 2015-11-29

**Authors:** Elijah Paintsil, Lorna Renner, Sampson Antwi, Joycelyn Dame, Anthony Enimil, Angela Ofori-Atta, Amina Alhassan, Irene Pokuaa Ofori, Xiangyu Cong, Tassos Kyriakides, Nancy R. Reynolds

**Affiliations:** ^a^ Departments of Pediatrics, Pharmacology & Public Health, Yale School of Medicine, New Haven, CT, USA; ^b^ Department of Child Health, University of Ghana School of Medicine and Dentistry, Accra, Ghana; ^c^ Korle-Bu Teaching Hospital, Accra, Ghana; ^d^ Department of Child Health, School of Medical Sciences, Kwame Nkrumah University of Science and Technology, Kumasi, Ghana; ^e^ Komfo Anokye Teaching Hospital, Kumasi, Ghana; ^f^ Yale Center for Analytical Sciences, Yale School of Public Health, New Haven, CT, USA; ^g^ Yale School of Nursing, West Haven, CT, USA

**Keywords:** pediatric HIV, disclosure, knowledge, stigma, illness perception, bioecological systems theory

## Abstract

The majority of HIV-infected children in sub-Saharan Africa have not been informed of their HIV status. Caregivers are reluctant to disclose HIV status to their children because of concern about the child’s ability to understand, parental sense of guilt, and fear of social rejection and isolation. We hypothesized that the low prevalence of pediatric HIV disclosure in Ghana is due to lack of accurate HIV information and high HIV stigma among caregivers. This is a preliminary analysis of baseline data of an HIV pediatric disclosure intervention study in Ghana (“Sankofa”). “Sankofa” – is a two-arm randomized controlled clinical trial comparing disclosure intervention plus usual care (intervention arm) vs usual care (control arm) at Korle-Bu Teaching Hospital (KBTH; control arm) and Komfo-Anokye Teaching Hospital (KATH; intervention arm). We enrolled HIV-infected children, ages 7–18 years who do not know their HIV status, and their caregivers. Baseline data of caregivers included demographic characteristics; Brief HIV Knowledge Questionnaire (HIV-KQ-18); Brief Illness Perception Questionnaire; and HIV Stigma Scale. Simple and multivariable linear regression analyses were used to assess the relationship between caregiver characteristics and HIV knowledge, stigma, and illness perception. Two hundred and ninety-eight caregivers were enrolled between January 2013 and July 2014 at the two study sites; KBTH (*n* = 167) and KATH (*n* = 131). The median age of caregivers was 41 years; 80.5% of them were female and about 60% of caregivers were HIV-positive. Seventy-eight percent of caregivers were self-employed with low household income. In both unadjusted and adjusted analyses, HIV negative status and lower level of education were associated with poor scores on HIV-KQ. HIV positive status remained significant for higher level of stigma in the adjusted analyses. None of the caregiver’s characteristics predicted caregiver’s illness perception. Intensification of HIV education in schools and targeted community campaigns are needed.

## Introduction

As antiretroviral therapy (ART) becomes increasingly available to children in resource-limited settings, millions of children infected with HIV are now expected to survive childhood through adolescence and into adulthood. Disclosure of HIV status to children with HIV is globally recognized as pivotal; disclosure is understood to confer a number of benefits affirmed by several studies and recommended by clinicians and leading pediatric treatment guidelines (Anttila, Metsahonkala, & Sillanpaa, 1999; Mellins et al., 2002; WHO, 2011). Disclosure of HIV status to children with HIV is beneficial to the child’s physical and psychological well-being, and improves both social functioning and clinical outcomes (Bachanas et al., 2001; Ferris et al., 2007; Lester et al., 2002; Menon, Glazebrook, Campain, & Ngoma, 2007). Moreover, the most important and predominant factor that negatively affects ART adherence in sub-Saharan Africa is lack of disclosure of HIV status (Nabukeera-Barungi, Kalyesubula, Kekitiinwa, Byakika-Tusiime, & Musoke, 2007). In addition, HIV-infected children in Ghana who knew their HIV positive status were more likely to take charge of taking their daily HIV medications (Kallem, Renner, Ghebremichael, & Paintsil, 2011)*.*

The fact that a majority of HIV-infected children in sub-Saharan Africa have not been informed of their HIV status (Vreeman, Gramelspacher, Gisore, Scanlon, & Nyandiko, 2013) reflects the resistance of both providers and caregivers. Caregivers are reluctant to disclose HIV status to their children because of concern about the child’s ability to understand, but also for fear of social rejection and isolation, parental sense of guilt, as well as fear that the child will not keep the diagnosis to themselves (Wiener, Mellins, Marhefka, & Battles, 2007), which is not the case with other chronic diseases such as childhood malignancy or cystic fibrosis (Bibace & Walsh, 1980; Slavin, O'Malley, Koocher, & Foster, 1982). Our previous work had shown that caregivers have a clear preference for assistance with disclosure from a health care provider (Kallem et al., 2011).

Based on these findings, we developed an HIV pediatric disclosure intervention model that is based on the bioecological systems theory, and core elements of the Information, Motivation, and Behavioral Skills (IMB) model of Health Behavior Change (Fisher & Fisher, 1992) to promote caregiver-to-child HIV disclosure. The intervention has two main components: (1) the use of an adherence and disclosure specialist (ADDS). The ADDS is familiar with the sociocultural norms of the community, and is trained to assist families in the process of disclosure (i.e., pre-disclosure, disclosure, and post-disclosure phases). Having a specific point person allows uniformity in approach and fosters development of a trusting relationship with the caregiver and child; and (2) disclosure as a process whereby the ADDS guides the intervention sessions to the IMB skills needs of the caregiver and the neurocognitive development of the child.

We hypothesized that the low prevalence of pediatric HIV disclosure in Ghana (Kallem et al., 2011) is due to lack of accurate HIV information, motivation, and disclosure skills of the caregivers. In this paper, we present survey data collected in Ghana on the knowledge and beliefs of caregivers (e.g., parents and guardians) of HIV-infected children who have not had their HIV status disclosed to them.

## Methods

### Study design and population

The Bioecological Pediatric HIV disclosure intervention study – “Sankofa” – is a two-arm randomized controlled clinical trial comparing treatment plus usual care (disclosure intervention arm) vs usual care alone (control arm). The study is ongoing in the two main teaching hospitals in Ghana: Korle-Bu Teaching Hospital (KBTH; control arm) and Komfo-Anokye Teaching Hospital (KATH; intervention). We randomized the sites to avoid cross-contamination within the same hospital, where the disclosure intervention administered to the intervention group could potentially filter into the control group (Levin, 2005a, 2005b). All HIV-infected children receiving care at the two teaching hospitals ages from 7 to 18 years who were started on ART within 12 months of study enrollment and who do not know their HIV status (based on caregiver account and medical records confirmation); and their caregivers were eligible for the study. Exclusion criteria included: HIV-infected children less than 7 years of age; children with congenital or developmental disorders, with comorbidities such as sickle cell disease or diabetes that require frequent clinic visits or hospitalizations, with AIDS-defining illness or end stage AIDS regardless of age. Written informed consent and assent was obtained from the caregivers and the children respectively. This study was approved by Institutional Review Boards of University of Ghana Medical School, Komfo Anokye Teaching Hospital, and Yale University Human Investigation Committee.

### Procedures

At enrollment, baseline data on the children and their caregivers were collected using validated study questionnaires. Each child–caregiver dyad is followed every three months post-enrollment through 24 months post-disclosure. Data collected at each visit include HIV disclosure status (child) and time of disclosure (if disclosed); IMB skills of caregiver. The content of each visit differs according to the site. At the disclosure intervention plus usual care site (KATH), the child–caregiver dyad meets with an ADDS to assist families go through the process of disclosure at each visit. After disclosure, the ADDS continues to meet with the caregiver and child at frequent and scheduled intervals for at least 24 months post-disclosure to assess post-disclosure problems, coping, and provide referral to services that the family may need. To make sure that the rate of disclosure at the intervention site is not confounded by time/attention of the ADDS, in the control hospital (KBTH), we also employ an ADDS specialist who meets with the caregivers but only provides general health information (e.g., nutritional) and answers questions the caregivers may have.

### Study measurements

The following measures of caregiver’s knowledge, motivation, and behavioral skills were assessed at baseline and follow-up: (1) Social Provisions Scale (SPS; Cutrona & Russell, 1987). This is a 24-item scale that entails six dimensions of support (social attachment, social integration, reassurance of worth, reliable alliance with others, guidance, and social nurturance) as well as an overall social support index. Its reliability and validity are supported by a large number of studies (Cutrona, 1989), including health intervention research in HIV-infected individuals (Kelly, Murphy, Bahr, et al., 1993; Kelly, Murphy, Sikkema, & Kalichman, 1993). Each of the 24 items has a possible value of 1–4; the possible range of the entire scale is 24–96; (2) Brief HIV Knowledge Questionnaire (HIV-KQ-18; Carey & Schroder, 2002), an 18-item measure with good psychometric properties which provides a measure of knowledge about HIV transmission. Correct answer is scored at 1 and a wrong answer or do not know answer is 0; the possible range of the entire scale is 0–18; (3) Brief Illness Perception Questionnaire (Brief IPQ; Broadbent, Ellis, Thomas, Gamble, & Petrie, 2009), a 9-item scale, which provides an assessment of the caregiver’s cognitive and emotional representations of HIV. Each of first 8 items has a possible value of 0–10. To compute the IPQ overall score, scores for items 3, 4, and 7 are reversed and added to the scores of items 1, 2, 5, 6, and 8. The possible range of IPQ score is 0–80. The last item (Item 9) is an open-ended question and does not contribute to the overall score; and (4) 18-item HIV Stigma Scale*,* a measure of caregiver perceptions of stigma related to HIV (Berger, Ferrans, & Lashley, 2001). Each of the 18 items has a possible value of 1–3; the possible range of score is 18–54. These instruments have been used in resource-limited settings (Kotze, Visser, Makin, Sikkema, & Forsyth, 2013; Lipps et al., 2010).

### Other explanatory variables

Additional variables are assessed at the baseline and follow-up study visits: age, marital status, sex, race/ethnicity, employment status, household income, education, duration and severity of illness, other siblings or family members living with HIV infection, ART treatment regimen, relationship to the child (e.g., biologic parents, family adopted parents, or unrelated adopted parents), perceived barriers to disclosure, optimum age for disclosure, and perceived advantages and disadvantages of disclosure.

### Data management

A Web-based data capture system is used for the acquisition, storage, and exploration of clinical and analytical data. The Sankofa database was established at cceHUB (http://ccehub.org), which has interactive interfaces to support the medical research workflow. Study participants are de-identified by auto-assigned alphanumeric identifiers. All electronic personal health information (ePHI) are encrypted for storage and decrypted on the fly for shared access and viewing. The medical research databases at cceHUB provide the US Health Insurance Portability and Accountability Act alignment for user authorization, access establishment, monitoring, audit, ePHI authentication, data integrity, backup, and disaster recovery.

### Statistical analysis

Overall and by study arm (i.e., hospital) demographic and other baseline data for the caregivers are presented. Means are provided for continuous variables as appropriate. Estimates of variability (standard deviations) are also provided for such variables. Numbers and percentages are provided for categorical variables. Simple and multivariable linear regression analyses were used to assess the relationship between caregiver characteristics and HIV Knowledge, stigma, and illness perception. All analysis and reports were generated using SAS v9.3, SAS Inc.

## Results

### Baseline demographic and social characteristics of caregivers

Two hundred and ninety-eight caregivers were enrolled from January 2013 to July 2014 at the two study sites; KBTH (*n* = 167) and KATH (*n* = 131). Table 1 illustrates the demographic and social characteristics of the caregivers from both sites. The mean age was 42.6 ± 10.4 years; majority of caregivers were female. About 60% of caregivers identified themselves as being HIV-positive. A little over half of the caregivers were married at enrollment. Fifty-six percent of the caregivers had an elementary education and about 16% had no formal education. Seventy-eight percent of caregivers were self-employed and over two-thirds of caregivers had a monthly household income of ≤300 Ghana Cedis (GHS) (equivalent to 94.3 USD). Although, most of the caregivers had less number of years at school and belonged to a lower income bracket, they accessed government health facilities for their health care needs.

**Table 1.** Characteristics of caregivers enrolled from January 2013 to July 2013.

	Komfo-Anokye (disclosure intervention)	Korle-Bu (usual care)		Total
	*N* = 131(%)	*N* = 167(%)	*P* value	*N* = 298(%)
Age^e^	43.2 ± 10.8	42.1 ± 10.0	0.34^a^	42.6 ± 10.4
Marital status^c^			0.09^b^	
Single	17 (13.0)	22 (13.2)		39 (13.1)
Married	63 (48.1)	98 (58.7)		161 (54.0)
Divorce/separated	17 (13.0)	23 (13.8)		40 (13.4)
Widowed	34 (26.0)	24 (14.4)		58 (19.5)
Gender			0.38^b^	
Male	22 (16.8)	36 (21.6)		58 (19.5)
Female	109 (83.2)	131 (78.4)		240 (80.5)
HIV status			0.9^b^	
No or not sure	53 (40.8)	65 (39.6)		118 (40.1)
Yes	77 (59.2)	99 (60.4)		176 (59.9)
Education			<0.0001^b^	
No school	17 (13.0)	30 (18.0)		47 (15.8)
Elementary education	96 (73.3)	71 (42.5)		167 (56.0)
Secondary and post-secondary education	18 (13.7)	66 (39.5)		84 (28.2)
Employment status			0.62^b^	
Unemployed	18 (13.8)	22 (13.3)		40 (13.5)
Self-employed	103 (79.2)	127 (76.5)		230 (77.7)
Private or government sector employment	9 (6.9)	17 (10.2)		26 (8.8)
Monthly household income (GHS)^d^			<0.0001^b^	
>50 per month	26 (20.2)	2 (1.2)		28 (9.6)
50–300 per month	84 (65.1)	99 (60.7)		183 (62.7)
>300 per month	19 (14.7)	62 (38.0)		81 (27.7)
Distance from clinic/hospital			0.0001^b^	
0–20 kilometers	98 (75.4)	90 (53.9)		188 (63.3)
More than 20 kilometers	32 (24.6)	77 (46.1)		109 (36.7)
Receiving own care at			0.56^b^	
Clinic/hospital	126 (96.9)	165 (98.8)		291 (98.0)
Clinic/hospital and Traditional/homeopathic healer	2 (1.5)	1 (0.6)		3 (1.0)
Other	2 (1.5)	1 (0.6)		3 (1.0)

^a^ *p* value was based on two sample *t test.*

^b^ *p* value was based on Fisher’s Exact test for count.

^c^Frequency and Column Percentage for categorical variables.

^d^Ghana Cedis (GHS): 1 GHS = 0.31 USD.

^e^Mean ± Standard deviation for continuous variable.

**Table 2.** Caregiver responses on Social Provision, HIV knowledge, Illness Perception and HIV stigma questionnaires.

	KATH^a^	KBTH^b^		Total
Questionnaire	*N* = 131	*N* = 167	*P* value^c^	*N* = 298
SPS				
Attachment (4–16)^e^	11.8 ± 1.8^d^	12.0 ± 2.4		11.9 ± 2.1
Social integration (4–16)	11.8 ± 1.2	12.3 ± 2.5		12.1 ± 2.1
Reassurance of worth (4–16)	11.6 ± 1.3	11.9 ± 2.6		11.8 ± 2.1
Reliable alliance (4–16)	11.4 ± 1.9	10.3 ± 3.6		10.8 ± 3.1
Guidance (4–16)	11.7 ± 1.7	12.2 ± 3.1		12.0 ± 2.5
Opportunity for nurturance (4–16)	12.6 ± 1.9	13.2 ± 2.5		12.9 ± 2.3
Overall SPS score (24–96)	70.9 ± 6.4	71.8 ± 10.4	0.34	71.4 ± 8.9
Illness perceptions (Brief IPQ)				
Consequences (0–10)	7.3 ± 3.1	7.2 ± 3.5		7.3 ± 3.3
Time line (0–10)	6.8 ± 2.7	5.7 ± 3.7		6.2 ± 3.4
Personal control (0–10)	7.2 ± 2.8	6.9 ± 3.6		7.1 ± 3.3
Treatment control (0–10)	9.4 ± 1.7	8.5 ± 2.7		8.9 ± 2.4
Identity (0–10)	1.7 ± 2.3	1.9 ± 2.5		1.8 ± 2.4
Illness concern (0–10)	7.5 ± 3.2	8.3 ± 3.0		7.9 ± 3.1
Coherence (0–10)	7.7 ± 2.4	7.2 ± 3.5		7.4 ± 3.1
Emotional (0–10)	5.4 ± 3.4	6.3 ± 3.9		5.9 ± 3.7
IPQ score (0–80)	34.1 ± 10.9	36.4 ± 11.7	0.09	35.4 ± 11.4
HIV stigma score				
Overall HIV stigma score (18–54)	41.2 ± 4.7	44.7 ± 6.1	<0.0001	43.2 ± 5.8
HIV-KQ-18				
Overall HIV-KQ-18 Score (0–18)	14.4 ± 2.1	13.6 ± 2.3	<0.01	13.9 ± 2.3

^a^KATH: Komfo-Anokye (disclosure intervention).

^b^KBTH: Korle-Bu (usual care).

^c^ *p* value was based on two sample *t-test*

^d^Mean, standard deviation.

^e^Possible range for each score.

### Caregivers' performance on SPS, Brief IPQ, HIV Stigma Scale, and HIV-KQ

The mean score for caregivers on the SPS was 71.4 out of a possible score range of 24–96 (Table 2). The mean score on the Brief IPQ for our caregivers was 35.4 ± 11.4 out of a highest possible score of 80. However, on two of the items they provided a more threatening score of either 9 or 10. These items were: “item 1” – How much does your child’s HIV affect your life? And “item 6” – How concerned are you about your child’s HIV?

The mean score of caregivers on the HIV Stigma Scale was 43.2 ± 5.8 out of a possible score range of 18–54; higher scores reflecting higher level of perceived stigma. The mean score of caregivers on the brief HIV-KQ-18 was 13.9 ± 2.3 out of a score range of 0–18. Although the overall score was high, there were inconsistencies in the performance across items (Table 3). None of the items was scored correctly by all caregivers. Five items were scored correctly by ≥90% of caregivers; 6 items were correctly scored by ≥80% of caregivers. Almost half of the caregivers gave incorrect responses to three items. These items were: “item 9” – “people are likely to get HIV by deep kissing, putting their tongue in their partner’s mouth, if their partner has HIV”; “item 12” – “a natural skin condom works better against HIV than does a latex condom”; and “item 18” – “using Vaseline or baby oil with condoms lowers the chance of getting HIV.”

**Table 3.** Performance on HIV-KQ-18 Questionnaire by caregivers.

Item	Content of HIV-KQ-18 Questionnaire	Correct response	Percent correct response
1	Coughing and sneezing DO NOT spread HIV.	T	82
2	A person can get HIV by sharing a glass of water with someone who has HIV.	F	94
3	Pulling out the penis before a man climaxes/cums keeps a woman from getting HIV during sex.	F	62
4	A woman can get HIV if she has anal sex with a man.	T	89
5	Showering, or washing one’s genitals/private parts, after sex keeps a person from getting HIV.	F	93
6	All pregnant women infected with HIV will have babies born with AIDS.	F	81
7	People who have been infected with HIV quickly show serious signs of being infected.	F	76
8	There is a vaccine that can stop adults from getting HIV.	F	80
9	People are likely to get HIV by deep kissing, putting their tongue in their partner’s mouth, if their partner has HIV.	F	51
10	A woman cannot get HIV if she has sex during her period.	F	86
11	There is a female condom that can help decrease a woman’s chance of getting HIV.	T	91
12	A natural skin condom works better against HIV than does a latex condom.	F	45
13	A person will NOT get HIV if she or he is taking antibiotics.	F	80
14	Having sex with more than one partner can increase a person’s chance of being infected with HIV.	T	94
15	Taking a test for HIV one week after having sex will tell a person if she or he has HIV.	F	69
16	A person can get HIV by bathing or swimming in a pool/river with a person who has HIV.	F	93
17	A person can get HIV from oral sex.	T	78
18	Using Vaseline or baby oil with condoms lowers the chance of getting HIV.	F	51

T, True; F, false.

## Predictors of caregivers’ HIV knowledge, illness perception, and HIV stigma perception

Univariate simple linear regression analyses were employed to assess characteristics of the caregiver such as age, gender, HIV infection status, level of education, monthly household income, or distance of caregiver’s home from a health facility that will predict caregiver’s HIV knowledge, illness perception, and HIV stigma perception. All the predictor variables that were associated with HIV knowledge, HIV stigma, and illness perception at the 0.1 level in the univariate analyses were included in the final multivariable regression model.

In the unadjusted analyses, HIV negative status (*P* < .0001) and low level of education (*P* < 0.001) were significantly associated with low scores on HIV knowledge scale (Table 4). There was no statistically significant association between gender, age, monthly income, distance from health facility, frequency of visits to health center and scores on HIV-KQ. In the adjusted model, HIV negative status (*P* < 0.001) and no formal school or low level of education (*P* < 0.0001) remained significantly associated with low level of HIV knowledge (Table 4). Caregivers who were HIV positive had statistically significant (*P* < 0.005) higher perception of HIV stigma (Table 5). However, only HIV positive status (*P* = 0.03) remained significantly associated with high level of HIV stigma perception in the adjusted model. We found no statistically significant association between any of the predictor variables and caregiver’s HIV illness perception in both unadjusted and adjusted models (Table 6).

**Table 4.** Unadjusted and adjusted linear regression analysis for caregivers scores on HIV-KQ.

	Unadjusted			Adjusted		
Characteristics	Estimates	Standard error	*P* value	Estimates	Standard error	*P* value
Age	−0.02	0.01	0.07	0.01	0.01	0.56
Gender						
Female	−0.49	0.33	0.13	–0.46	0.33	0.16
Male (reference group)						
HIV status						
Yes	0.90	0.26	0.0006	0.91	0.28	0.0014
No (reference group)						
Education			<0.0001^a^			<0.0001^a^
Secondary and post-secondary education	1.92	0.40	<0.0001	1.80	0.42	<0.0001
Elementary education	1.60	0.36	<0.0001	1.53	0.36	<0.0001
No school (reference group)						
Monthly household income (GHS)^b^			0.11^a^			0.28^a^
Greater than 300 per month	0.53	0.49	0.28	0.18	0.48	0.72
50–300 per month	−0.09	0.46	0.84	−0.27	0.44	0.54
Less than 50 per month (reference group)						
Distance from clinic/hospital						
More than 20 kilometers	−0.20	0.27	0.46	−0.25	0.26	0.33
0–20 kilometers (reference group)						

^a^Type 3 overall *p* value for categorical variable with more than 2 levels.

^b^Ghana Cedis (GHS): 1 GHS = 0.31 USD.

**Table 5.** Unadjusted and adjusted linear regression analysis for caregivers scores on HIV stigma questionnaire.

	Unadjusted			Adjusted		
Characteristics	Estimates	Standard error	*P* value	Estimates	Standard error	*P* value
Age	−0.05	0.03	0.1122	0.01	0.04	0.80
Gender						
Female	0.64	0.85	0.45	0.35	0.91	0.70
Male (reference group)						
HIV Status						
Yes	1.95	0.68	0.005	1.68	0.79	0.03
No (reference group)						
Education			0.71^a^			0.80^a^
Secondary and post-secondary education	0.34	1.06	0.75	−0.22	1.15	0.85
Elementary education	−0.31	0.96	0.75	−0.58	0.99	0.56
No school (reference group)						
Monthly household income (GHS)^b^			0.07^a^			0.15^a^
Greater than 300 per month	2.74	1.28	0.03	2.54	1.33	0.06
50–300 per month	1.48	1.19	0.21	1.51	1.21	0.21
Less than 50 per month (reference group)						
Distance from clinic/hospital						
More than 20 kilometers	1.26	0.70	0.07	0.92	0.72	0.20
0–20 kilometers (reference group)						

^a^Type 3 overall *p* value for categorical variable with more than 2 levels.

^b^Ghana Cedis (GHS): 1 GHS = 0.31 USD.

**Table 6.** Unadjusted and adjusted linear regression analysis for caregivers scores on HIV illness perception questionnaire.

	Unadjusted			Adjusted		
Characteristics	Estimates	Standard error	*P* value	Estimates	Standard error	*P* value
Age	0.02	0.06	0.70	0.05	0.08	0.48
Gender						
Female	0.64	1.67	0.70	1.46	1.84	0.43
Male (reference group)						
HIV status						
Yes	−0.55	1.37	0.69	−0.66	1.59	0.68
No (reference group)						
Education			0.86^a^			0.98^a^
Secondary and post-secondary education	0.54	2.08	0.80	0.40	2.33	0.86
Elementary education	−0.32	1.89	0.87	0.17	2.02	0.93
No school (reference group)						
Monthly household income (GHS)^b^			0.31^a^			0.36^a^
Greater than 300 per month	3.17	2.55	0.21	3.43	2.70	0.20
50–300 per month	1.15	2.36	0.63	1.59	2.45	0.52
Less than 50 per month (reference group)						
Distance from clinic/hospital						
More than 20 kilometers	1.93	1.37	0.16	1.88	1.45	0.20
0–20 kilometers (reference group)						

^a^Type 3 overall *p* value for categorical variable with more than 2 levels.

^b^Ghana Cedis (GHS): 1 GHS = 0.31 USD.

## Discussion

The bioecological Pediatric HIV disclosure intervention trial is ongoing at two sites in Ghana (Accra and Kumasi). The concept is grounded in the traditional belief of the “Akan” tribe of Ghana of the need for critical and analytical review of the past to inform present policies and practices (“Sankofa”; literally translated to mean “go and retrieve”) and the bioecological systems theory. In this fluid context, we sought to assess whether baseline characteristics of caregivers who have not disclosed the HIV status of their children will predict their HIV knowledge, HIV-related stigma and illness perception. We found that most of the caregivers had inaccurate HIV transmission knowledge and high level of perceived HIV stigma. Furthermore, HIV positive status and low level of formal education were significantly associated with HIV knowledge and stigma perception. These predictor variables could be modified through the interaction of caregivers with the Sankofa study ADDS. The ADDS will help caregivers to consolidate their knowledge and clarify any misconceptions. Previous studies on pediatric HIV disclosure reported that caregivers with higher level of education were more likely to disclose the HIV status to their children (Biadgilign, Deribew, Amberbir, Escudero, & Deribe, 2011; De Baets, Sifovo, Parsons, & Pazvakavambwa, 2008; Vreeman et al., 2013). The caregivers enrolled in Sankofa have not disclosed the HIV status of the children to them; our finding of low level of education among our caregivers is consistent with that of previous studies. It is possible that HIV education was not part of the curriculum when most of the caregivers were in elementary school about 20 years ago. Interestingly, school enrollment and higher level of education of HIV-infected children have been associated with HIV status disclosure (Bhattacharya, Dubey, & Sharma, 2011; Kallem et al., 2011; Myer, Moodley, Hendricks, & Cotton, 2006; Vaz et al., 2011).

In most sub-Saharan African countries, HIV transmission and prevention campaigns are carried out at the community level on routine basis. The inaccuracies in responses given by our caregivers, particularly on the use of condoms to prevent HIV (Table 3), may be a reflection of caregivers understanding of the content of HIV health education received. Thus health literacy, defined as the ability to read and understand medical information, may be a key factor in the disclosure of HIV status to HIV-infected children in resource-limited settings. Health literacy has been associated with favorable HIV treatment outcomes (Kalichman, Ramachandran, & Catz, 1999; Kalichman & Rompa, 2000; Kalichman et al., 2000; Weiss et al., 2003). Although, the instruments we used have been validated in some resource-limited countries (Kotze et al., 2013; Lipps et al., 2010), consideration should be given to validating them in the country of study prior to their use. For instance, it was anticipated that some participants, given low levels of education, would have difficulty completing study questionnaires independently from pre-testing of the instruments prior to study inception. This was handled by collecting the data orally in interviews. However, we did not anticipate that some participants will have difficulty rendering responses in quantitative Likert-type scales (range 1–4). This has been handled by having the interviewers use a culturally familiar, pictorial aid such as baskets filled to different levels to signify a little or a lot of agreement with the questionnaire statement. On the other hand, health literacy alone may not be sufficient to affect behavior (Anderson, 1996; Nicholson, Mellins, Dolezal, Brackis-Cott, & Abrams, 2006). Self-efficacy, confidence in one’s ability to carry out certain behaviors, has been associated with adherence to treatment in several chronic diseases (Johnson et al., 2003; Kavanagh, Gooley, & Wilson, 1993; Reynolds et al., 2004; Rhodes, Martin, & Taunton, 2001). The role of self-efficacy in disclosure of HIV status to children in resource-limited settings is understudied. Our study aims at using ADDS to help caregivers develop the skills needed to be able to disclose to their children.

The fear of stigma after disclosure of the HIV status to a child has been the most formidable barrier to disclosure (Bhattacharya et al., 2011; Biadgilign et al., 2011; Kallem et al., 2011; Vreeman et al., 2010). Our cohort of caregivers who have not disclosed the HIV status to their children had higher scores on the HIV Stigma Scale. Of note, caregivers who were HIV-infected had higher perception of HIV stigma (*P* < 0.005) compared to caregivers who were HIV-uninfected. HIV stigma devalues and discriminates against persons living with HIV/AIDS or those associated with them and stems from the underlying stigmatization of modes of acquisition of HIV (Abell, Rutledge, McCann, & Padmore, 2007; Herek, Gillis, & Cogan, 1999). It is conceivable that by not disclosing the status to these children the caregivers believe that they are protecting their love ones from an unfair and an unjust treatment from the society.

In conclusion, level of education, health literacy, and HIV associated stigma are challenging barriers to pediatric HIV disclosure; however, they can be modified in well-designed HIV education campaigns. The findings from Sankofa study may inform the content of such programs.
